# Punctuation and Implicit Prosody in Silent Reading: An ERP Study Investigating English Garden-Path Sentences

**DOI:** 10.3389/fpsyg.2016.01375

**Published:** 2016-09-15

**Authors:** John E. Drury, Shari R. Baum, Hope Valeriote, Karsten Steinhauer

**Affiliations:** ^1^Department of Linguistics, Stony Brook UniversityNew York, NY, USA; ^2^School of Communication Sciences and Disorders, McGill UniversityMontreal, QC, Canada; ^3^Centre for Research on Brain, Language and MusicMontreal, QC, Canada; ^4^Glenrose Rehabilitation Hospital, Alberta Health ServicesEdmonton, AB, Canada

**Keywords:** commas, punctuation, English garden-path sentences, implicit prosody, closure positive shift (CPS), boundary deletion hypothesis, event-related potentials (ERP), silent reading

## Abstract

This study presents the first two ERP reading studies of comma-induced effects of covert (implicit) prosody on syntactic parsing decisions in English. The first experiment used a balanced 2 × 2 design in which the presence/absence of commas determined plausibility (e.g., *John, said Mary, was the nicest boy at the party* vs. *John said Mary was the nicest boy at the party*). The second reading experiment replicated a previous auditory study investigating the role of overt prosodic boundaries in closure ambiguities (Pauker et al., 2011). In both experiments, commas reliably elicited CPS components and generally played a dominant role in determining parsing decisions in the face of input ambiguity. The combined set of findings provides further evidence supporting the claim that mechanisms subserving speech processing play an active role during silent reading.

## Introduction

The strong influence of prosodic boundaries in guiding auditory language processing has been convincingly demonstrated through the use of various experimental paradigms (e.g., Kjelgaard and Speer, 1999), including online measures such as eyetracking (e.g., Hirotani et al., 2006; White et al., 2014) and event-related potentials (ERPs; e.g., Steinhauer et al., 1999). Since it was found that auditory prosodic boundaries can both immediately induce or avert garden-path effects in the processing of ambiguous German sentence structures, and that ERPs reflect these processes in real time, numerous ERP replications of the effect have been reported cross-linguistically (e.g., Kerkhofs et al., 2008; Mietz et al., 2008; Pauker et al., 2011; Bögels et al., 2013). Importantly, in addition to prosody-induced ERP garden path effects, Steinhauer et al. (1999) also identified a unique ERP component that is immediately evoked in response to the presence of a prosodic boundary: the closure positive shift (CPS).

Unlike speech, written language does not provide the same wealth of prosodic information. However, according to the *implicit prosody hypothesis* (IPH), introduced by Fodor (1998, 2002) and Bader (1998), even silent readers activate prosodic patterns, which then influence sentence processing further downstream. Unfortunately, these effects during silent reading are difficult to study, and reported effects have often been discussed controversially^1^. In 2001, a German reading study modeled after the auditory CPS study in 1999, found initial ERP evidence that commas disambiguate certain structural ambiguities in similar ways as overt prosodic speech boundaries, and—moreover—that they also trigger a CPS (Steinhauer and Friederici, 2001). Similar CPS components were also observed when readers were instructed to reproduce prosodic boundaries in specific positions while silently reading sentences, strongly suggesting that the comma-induced CPS was in fact related to prosodic phrasing (Steinhauer, 2003). In addition, when commas were used to induce inappropriate parsing decisions, the resulting ERP garden-path effects (N400s and P600s) were similar to those found for effects in the auditory modality induced by prosodic boundaries. The authors concluded that commas induce implicit prosodic boundaries which then have a similar impact on parsing decisions as overt boundaries.

In stark contrast to these results, Kerkhofs et al. (2008) failed to elicit a CPS in response to commas in temporarily ambiguous sentences in Dutch, although commas did influence parsing and a CPS did emerge in response to prosodic boundaries in *auditory* versions of the same stimuli (Kerkhofs et al., 2008). The sentence structures employed by Kerkhofs et al. (2008)—noun phrase (NP) vs. sentence (S) coordination—differed substantially from the early and late closure ambiguities examined by Steinhauer et al. (Steinhauer et al., 1999; Steinhauer and Friederici, 2001), potentially contributing to the different patterns of findings across the investigations. The strictness with which punctuation rules are applied in the two languages studied may also have influenced the outcomes. Kerkhofs et al. (2008) suggest that conscious attention to the punctuation may have contributed to the emergence of the CPS in (Steinhauer and Friederici's 2001) experiment, due to the presence of structural violations induced by the commas; in contrast, in their own experiment, the stimuli did not include violations of punctuation rules and thus did not result in conscious attention directed to the commas. However, in contradiction to this line of argument, Steinhauer (2003) had already published an ERP experiment on comma processing in German that did *not* include any (comma-induced) violations, or violations of comma rules, but still elicited a CPS. This suggests that the elicitation of the CPS does not depend on special attention related to violations of comma rules (contra Kerkhofs et al., 2008).

One other ERP investigation, in yet another language, directly explored the presence of CPS-like components in response to commas presented in Chinese stimuli and reported the consistent emergence of a CPS across three tasks (Liu et al., 2010). The nature of the stimuli and the influence of the comma were somewhat different from the other studies discussed above, making direct comparisons difficult.

While the specific relationship between punctuation and prosody remains controversial (see Chafe, 1988; Hill and Murray, 2000; Fodor, 2002; Steinhauer, 2003)—and the data gathered to date regarding elicitation of the CPS in response to commas are equivocal—there is ample reason to further explore the cognitive mechanisms underlying the processing of commas in reading ambiguous sentence structures. First and foremost, this kind of research is needed to identify both parallels and differences across processing modalities in sentence processing, and in particular to advance our understanding of the role of implicit and explicit prosody. In addition, there has not been a single ERP study investigating punctuation effects in English. The objective of the present investigation is precisely this—to examine how English readers use punctuation in real-time processing of ambiguous sentences.

To this end, we carried out an ERP study in English involving silent, word-by-word (RSVP) reading. Two paradigms were investigated. In the first, we used commas to modulate the relationships between proper names (e.g., *John, Mary*) and predicate nominals to either induce a gender mismatch (indicated by a “?”), as in (1b) compared to (1a), or to avoid one [in (1d) compared to (1c)].

(1) A. John said Mary was the nicest **girl** at the party.
            no commas / correct
     B. John, said Mary, was the nicest ?**girl** at the party.
            commas / violation
     C. Mary said John was the nicest ?**girl** at the party.
            no commas / violation
     D. Mary, said John, was the nicest **girl** at the party.
            commas / correct

In contrast to most ERP studies investigating the impact of (overt or covert) prosodic information on syntactic parsing decisions, this first paradigm presents a completely balanced 2 × 2 design (as recommended by Steinhauer and Drury, 2012) that allows inspection of how punctuation can guide parsing decisions in real-time. For condition C (as compared to condition A), the ERP literature would predict an N400 effect reflecting the detection of a conceptual-semantic anomaly, and we expect to find the same effect for condition B relative to D. That is, the insertion of commas (conditions B and D) should yield parenthetical readings where the first noun phrase (*John* in B and *Mary* in D) serves as the subject of the downstream predicate (*was the nicest girl*). Thus, as a consequence of the presence of the comma in B a semantic anomaly will arise at the target word of the same type as we see in C. In addition, we predict CPS components at all comma positions, if English readers use this information online to generate implicit boundaries.

In the second paradigm, tested in the same session and the same group of participants as (1), we examined the classic closure ambiguities illustrated in (2), using the identical materials employed in a previous auditory ERP study (see Pauker et al., 2011 for details).

(2) A. While the boy was browsing the book, the game slipped off the table.
     B. While the boy was browsing, the book slipped off the table.
     C. While the boy was browsing the book slipped off the table.
     D. While the boy was browsing, the book, the game slipped off the table.

Conditions A and B are correct control conditions with appropriate commas in an early position (Early closure condition B) or a late position (Late closure condition A). Conditions C and D are garden path conditions with a missing comma (in C as compared to B) or a superfluous comma (in D as compared to A). As in our first experiment above, commas at all positions are expected to elicit CPS components, if they trigger prosodic boundaries. Moreover, if commas influence English readers in similar ways as prosodic boundaries influence English listeners, then conditions C (without a required early comma) and D (containing a superfluous early comma) are expected to yield the same ERP responses found in the auditory domain. At the point where disambiguating information is encountered in condition C (i.e., the main clause verb *slipped*), Pauker et al. found a classic P600 garden-path effect (Osterhout and Holcomb, 1992; Steinhauer et al., 1997). This is consistent with a wealth of previous closure ambiguity findings (see Frazier, 1987) showing the tendency of parsing mechanisms to analyze the noun phrase following the first verb as its direct object (which, in the C condition, must then be reanalyzed as the subject of the main clause when the main clause verb is encountered). The fact that this effect arose in comparison of condition C to B shows that the prosodic boundary in B was sufficient to locally block that (ultimately incorrect) parse of the input. For the additional prosodic boundary in Condition D, in contrast, Pauker et al. found a biphasic N400/P600 pattern relative to condition A. This pattern was suggested to reflect a combination of argument structure processing difficulties and attempted repair/reanalysis (see Pauker et al., 2011 and also our Discussion below for further remarks about condition D). Generally: if these response profiles found in the auditory domain for condition C and D (relative to B and A, respectively) are generalizable across modalities, we should find them here as well.

## Methods

### Participants

Twenty six healthy monolingual English speakers (13 females) from McGill University with normal or corrected-to-normal vision participated in the experiment. All subjects were between the ages of 18 and 30 (average: 22.6 years, SD: 3.1 years) and were right-handed (as confirmed by the Edinburgh Handedness Inventory, Oldfield, 1971). Subjects were paid for their participation and provided written informed consent to participate prior to beginning the experiment.

### Stimuli

Two sets of stimuli were separately created for this study, one for each of the two paradigms introduced above. We will describe each in turn.

#### Parentheticals and gender mismatches

The stimuli for this study were of the four main types in (1A-D) above, constructed in pairs of 4-tuples generated by varying the gender of the predicate nominal (i.e., *girl/boy*), as in (3):

(3) A. John said Mary was the nicest **girl / ?boy** at the party.
            no commas / correct
     B. John, said Mary, was the nicest ?**girl / boy** at the party.
            commas / violation
     C. Mary said John was the nicest ?**girl / boy** at the party.
            no commas / violation
D. Mary, said John, was the nicest **girl / ?boy** at the party
            commas / correct

Conditions (A) and (C) consisted of two clauses, and differed only in whether the subject in the embedded clause [e.g., *Mary* in (3A), *John* in (3C)] mismatched with the subsequent head noun [e.g., …John *was the nicest* girl…in (3C)]. Conditions (B) and (D) were derived from (A) and (C), respectively, by including commas both after the main clause subject and after the embedded subject, keeping all else constant. The inclusion of the commas thus mapped the bi-clausal (A)/(C) examples to mono-clausal counterparts (B)/(D) with “said [proper-name]” introduced parenthetically. Thus, while *Mary* is the subject of the embedded clause in (3A), adding the two commas, as in (3B), makes *John* the subject connected to the predicate “was the nicest girl,” creating a gender mismatch. Conversely, the mismatch violation in (3C) disappears in (3D) with the inclusion of the commas, as this makes *Mary* the subject related to the predicate “was the nicest girl,” resulting in a gender match.

The items representing the four conditions (A)-(D) were all based on 16 masculine/feminine target word pairs (e.g., *boy/girl, king/queen*, etc.). A master list of 512 sentences was generated as follows based on sets of 8 matched sentences like those in (3). First, for each set of 8 sentences, another 8 matching cases were derived by replacing the two proper names [e.g., putting in *Fred*/*Sarah* for all corresponding occurrences of *John/Mary* in (1)/(2)]. This yielded sets of 16 matched sentences. Thirty-two such sets of 16 were generated to produce the master list of 512 items. This master list was then divided into four separate presentation lists of 128 items each [32 items for each condition (A)–(D) per list], such that proper-name pairs [e.g., *John/Mary* in (1) above] never occurred in the same order within a list, and never were repeated in the same condition. The occurrences of target word pairs (*boy/girl, king/queen*, etc.) were also evenly distributed across all conditions within each list. Items within given lists that belonged to the same matched set of 16 from the master list were assigned to separate quarters of the experiment.

Finally, the 128 experimental items were intermixed with the closure ambiguity paradigm stimuli (160 sentences, half with violations, explained below), and an additional 120 filler items (half of which contained violations) which were part of a separate experiment (not discussed here). The resulting 4 lists of 408 items each were then subjected to a pseudo-randomization procedure which evenly dispersed the various sentence types across the experiment and which also ensured that no similar sentence types occurred in direct succession. Presentation of the four lists was counterbalanced across participants.

#### Commas and closure ambiguity

This paradigm employed stimuli consisted of 4 main sentences types which varied with respect to Early Closure (EC) vs. Late Closure (LC) of the initial clause, as indicated by punctuation. Since these stimuli were written versions of auditory stimuli discussed in detail in Pauker et al. (2011), we will only describe them briefly here.

In all cases, the first verb phrase was headed by an optionally transitive verb so as to be consistent with both early and late closure. These verbs were kept in the progressive aspect (“*was browsing”*) to eliminate reported biases toward the transitive use in the past or present tense, while maintaining the possibility of a garden-path effect (Frazier et al., 2006). The verb phrases were either followed by an object noun phrase (NP) and then a second sentential clause (LC conditions 2A/D above), or directly by a second sentential clause (EC conditions 2B/C). Within the two types of sentences, A and D differ only in that D has a superfluous (early) comma (suggesting initial EC parsing instead of the required LC parsing), whereas C differs from B in that C lacks the early comma (suggesting initial LC parsing instead of the required EC parsing). Thus, differences between garden path conditions (C and D) and their correct controls (B and A, respectively) only depended on the presence or absence of commas as cues to prosodic boundaries.

As in Pauker et al. (2011), forty sets of A/B/C/D conditions were used. The 160 stimuli were divided into the four quarters of our presentation lists so that repetitions of any given 4-tuple were maximally separated. Further, the order in which each condition from a given 4-tuple appeared within a list was varied systematically across our four presentation lists (Latin Square).

### Procedure

Subsequent to a practice block of 8 trials, participants were presented with six blocks of 68 sentences (= 408 experimental items total) in a shielded, sound-attenuating chamber. Participants were required to provide sentence-final acceptability judgments by clicking on a mouse button. Each trial was constructed as follows. Prior to visual presentation of the first word of the sentence, a fixation cross was presented in the middle of the computer screen for 500 ms to alert subjects to the upcoming stimulus. Stimuli were presented word-by-word for 300 ms each, followed by a 200 ms blank screen. For stimuli which included commas, the comma was presented along with the word it followed. At the end of the sentence, subjects were prompted to provide the acceptability judgment by presentation of the word “Good?.” After the judgment, a visual cue (“!!!”) was provided indicating that subjects were encouraged to blink their eyes during that interval. Short breaks separated the six experimental blocks.

#### EEG recording

EEG was continuously recorded (500 Hz sampling rate; Neuroscan Synamps2 amplifier) from 19 cap-mounted Ag/AgCl electrodes (Electro-Cap International) referenced to the right mastoid and placed according to the 10–20 System (impedance < 5 Ω). EOG was recorded from bipolar electrode arrays.

#### Data processing and analysis

Acceptability judgment data were subjected to repeated-measures ANOVAs, separately for the two paradigms (factors Comma (presence/absence) × Correctness (good/bad) for the parentheticals), and the four level condition factor (A–D) for the Closure sentences [see (2) above].

EEG data were analyzed using the Matlab based platforms EEGLAB and ERPLAB. Single subject averages were computed for all conditions and time-locking points following data pre-processing which included filtering (0.1–30 Hz bandpass), detrending, and artifact rejection (carried out first automatically and then followed up with by hand inspection to ensure no artifacts were missed and no trials were erroneously excluded). Given our expected patterns are well-established in the literature (CPS, N400, and P600 effects) and have known prototypical onset-timing and scalp distributions (Friederici et al., 1999; Steinhauer and Connolly, 2008), we pursued a simple region of interest (ROI) analysis of the EEG data, collapsing anterior electrodes FP1/2, F3/4, and Fz into an anterior region, and posterior electrodes C3/4, P3/4, Cz, and Pz into a single posterior region. Condition factors from each of our two paradigms were thus examined together with this two level topographical factor in four successive 200 ms time-windows from 100 ms post-target-word onset to 900 ms (i.e., 100–300, 300–500, 500–700, and 700–900 ms). Mean amplitude was the dependent measure in all analyses, relative to a 200 ms pre-target word baseline (i.e., −200–0 ms).

## Results

### Sentence final acceptability judgments

**Figure 1:**
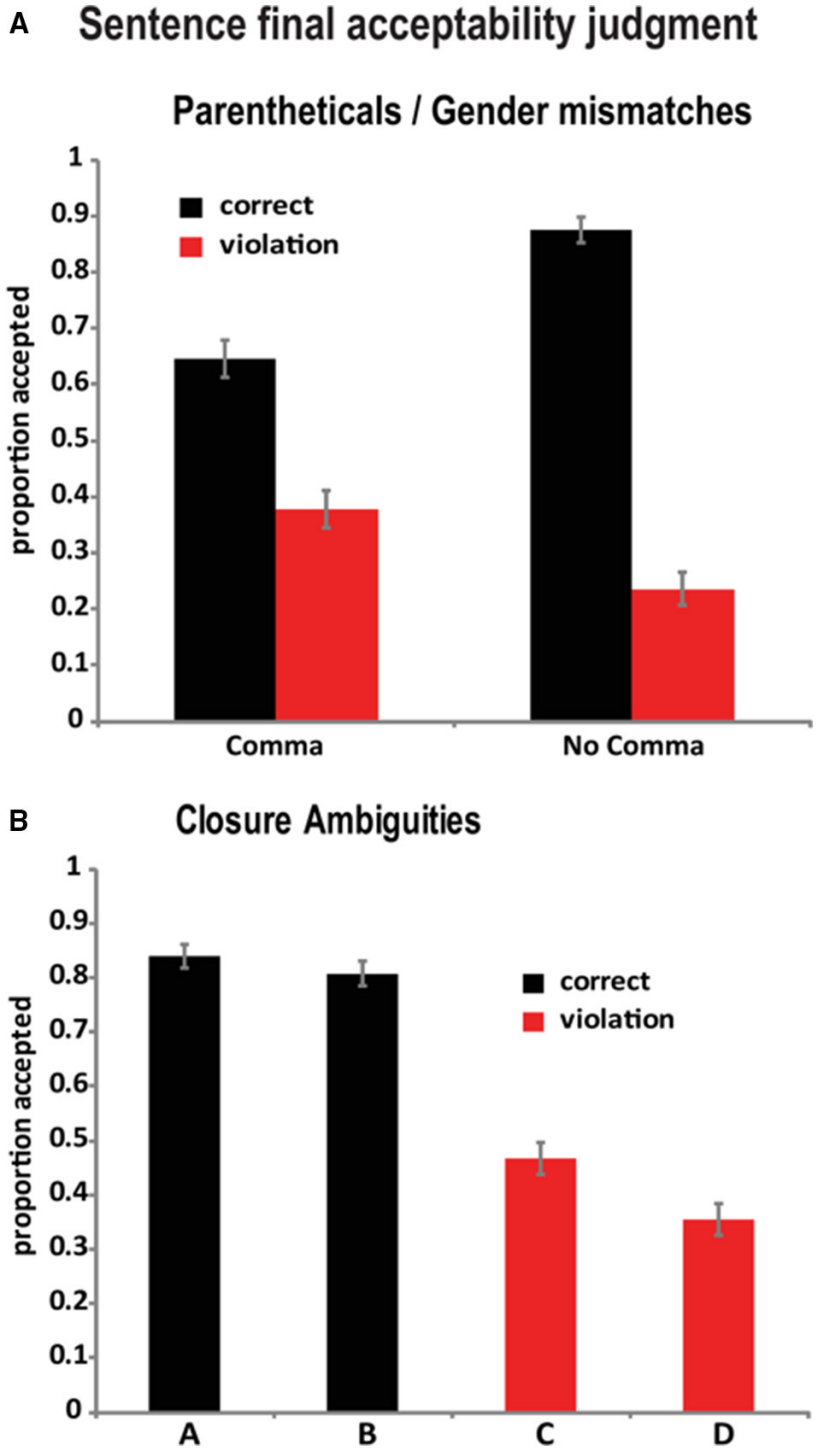
**Sentence final acceptability judgment data (proportion accepted) for (A) parentheticals and (B) closure ambiguities (error bars indicate ±95% confidence intervals)**.

Responses to our end of sentence judgment task are shown in Figure 1. For the parenthetical/gender-mismatch paradigm, readers reliably distinguished between correct and violation conditions [*F*_(1, 25)_ = 50.98, *p* < 0.0001], but this interacted with the presence/absence of commas [comma × correctness: *F*_(1, 25)_ = 12.86, *p* = 0.0014]. This interaction was due to the fact that although readers did reliably discriminate between the gender matches/mismatches when commas were present [*F*_(1, 25)_ = 7.43, *p* = 0.0116], they did so less robustly than when commas were absent [*F*_(1, 25)_ = 128.01, *p* < 0.0001].

Our closure sentences yielded a significant effect of Condition (A/B/C/D) [*F*_(3, 75)_ = 49.95, *p* < 0.0001], due to the reduced acceptance rates for the garden path (C) and the anomalous (D) condition. Pair-wise comparisons (Holm-Bonferroni corrected *p*-values) showed that though (A) obtained numerically higher acceptance rates than the (B) cases, this difference did not reach significance [*F*_(1, 25)_ = 2.65, *p* = 0.12]. In contrast, the deviant cases (C) and (D) did differ, with (D) significantly less acceptable than (C) [*F*_(1, 25)_ = 5.73, *p* = 0.0490].

### Event related potentials

#### Parentheticals and gender mismatches

**Figure 2:**
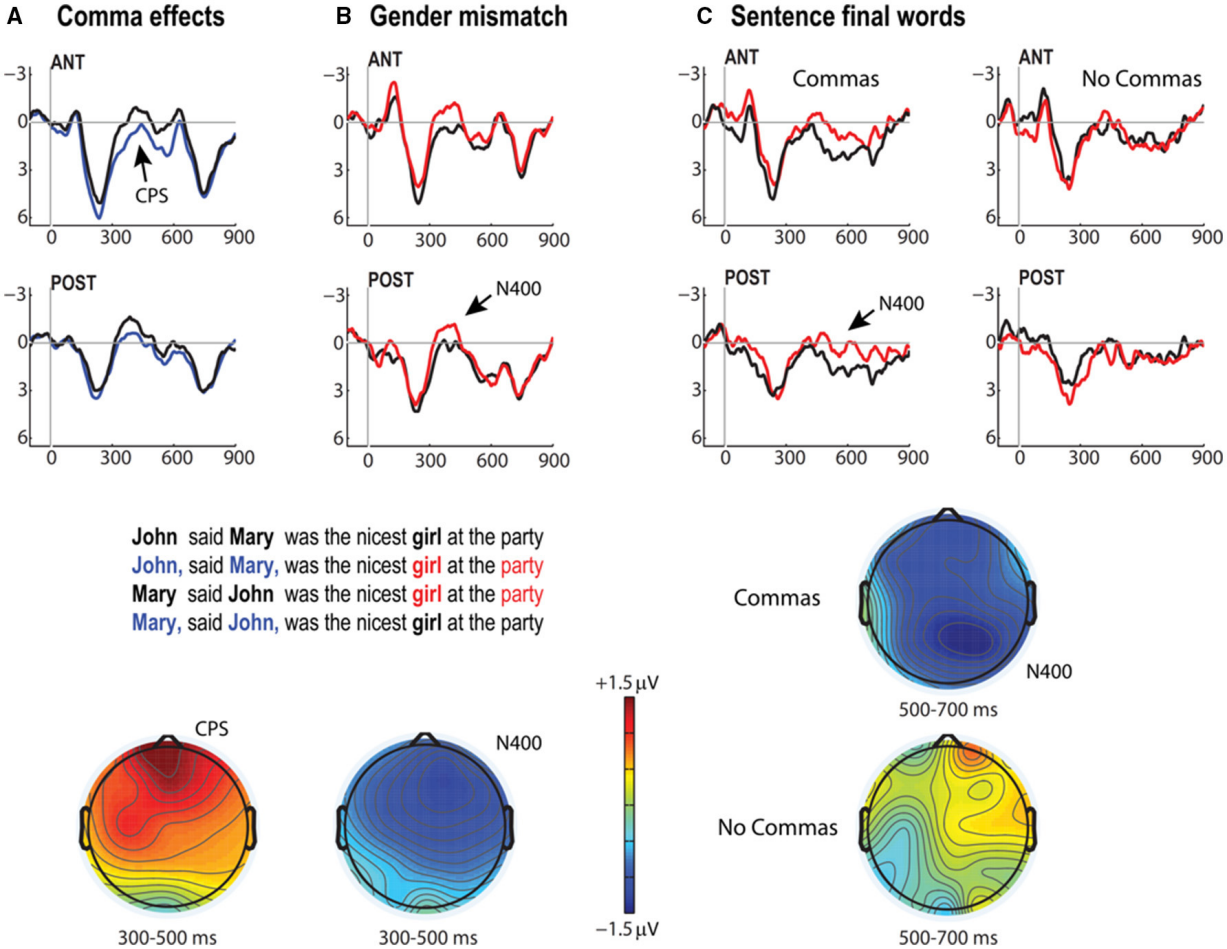
**CPS and N400 effects for the Parenthetical/Gender Mismatch Paradigm**. Grand average ERPs (*N* = 26) for anterior and posterior ROIs collapsed over: **(A)** all comma-present (blue) vs. comma-absent (black) position; **(B)** both gender mismatch (red) vs. both gender match conditions (black); sentence-final N400 effects are shown in **(C)**. Voltage maps show mean amplitude differences over all scalp electrodes for the CPS (comma-present minus comma absent) and both target-word and sentence-final word N400 effects (gender mismatch minus gender match), scale represents +1.5 μV (red) to −1.5 μV (blue).

We expected that commas in our parenthetical conditions would elicit CPS effects, and that gender mismatches between the proper names and the noun phrase predicate would elicit N400 effects. Both of these findings obtained (Figure 2). First, the presence of commas was attended by a relative positivity [see (A) in Figure 2] with an anterior maximum which began sometime in the first 100–300 ms time-window and persisted through both the 300–500 and 500–700 ms ranges. This gave rise to main effects of comma in all three time-windows [100–300 ms, *F*_(1, 25)_ = 4.91, *p* = 0.036; 300–500 ms, *F*_(1, 25)_ = 15.68, *p* = 0.0005; 500–700 ms, *F*_(1, 25)_ = 10.5, *p* = 0.0034]. There were no significant interactions between comma and the position of the proper names. There was, in the 300–500 ms time-window, a marginal interaction of comma with anterior/posterior (ap) [*F*_(1, 25)_ = 4.09, *p* = 0.054], reflecting the fact that this effect was maximal over the anterior ROI.

However, a concern regarding the early onset of the positive-going deflection elicited by commas raises the question of whether at least the early part of this effect (100–300 ms) may be just a modulation of the P2. But if this was so we would not expect the positivity to both continue in time and grow in amplitude. Further, as we will note below, the comma comparisons in our closure ambiguity manipulations did not demonstrate this early onset. To see whether the positivity in the 100–300 ms range could be distinguished from the 300–500 ms effect, we compared the time-windows directly. This analysis revealed a significant comma × time-window interaction [*F*_(1, 25)_ = 5.59, *p* = 0.026], suggesting an additional independent effect beyond the effect evident in the earliest 100–300 ms range (we return to this matter below in our Discussion).

The second predicted effect was connected to the gender mismatches, which elicited a clear N400 response [see (B) in Figure 2], yielding a main effect of correctness [*F*_(1, 25)_ = 6.91, *p* = 0.014] in the 300–500 ms time-window. Correctness did not interact with the presence/absence of upstream commas (*F*'s < 1). It is worth flagging here that the lack of this interaction is of interest, given the pattern evident in the sentence-final acceptability judgment task reported above (where it seems participants were less certain about the gender mismatches when they were induced by the presence of commas). One might have anticipated on the basis of the behavioral data a less consistent violation response profile for the comma cases. That is, whatever the source of the confusion that arose by sentence end, we see no antecedent of this in terms of online N400 responses.

In order to probe this matter further, we conducted an additional set of exploratory analyses of sentence final words in these stimuli. As can be seen in (C) (right hand plots in Figure 2), sentence-final words gave rise to a late N400-like effect, present only for the Comma conditions in the 500–700ṁs [*F*_(1, 25)_ = 10.24, *p* = 0.0038] and 700–900 ms time-windows [*F*_(1, 25)_ = 8.68, *p* = 0.0069]. This effect did not manifest in the No Comma conditions (*F*'s < 1).

#### Commas and closure ambiguities

**Figure 3:**
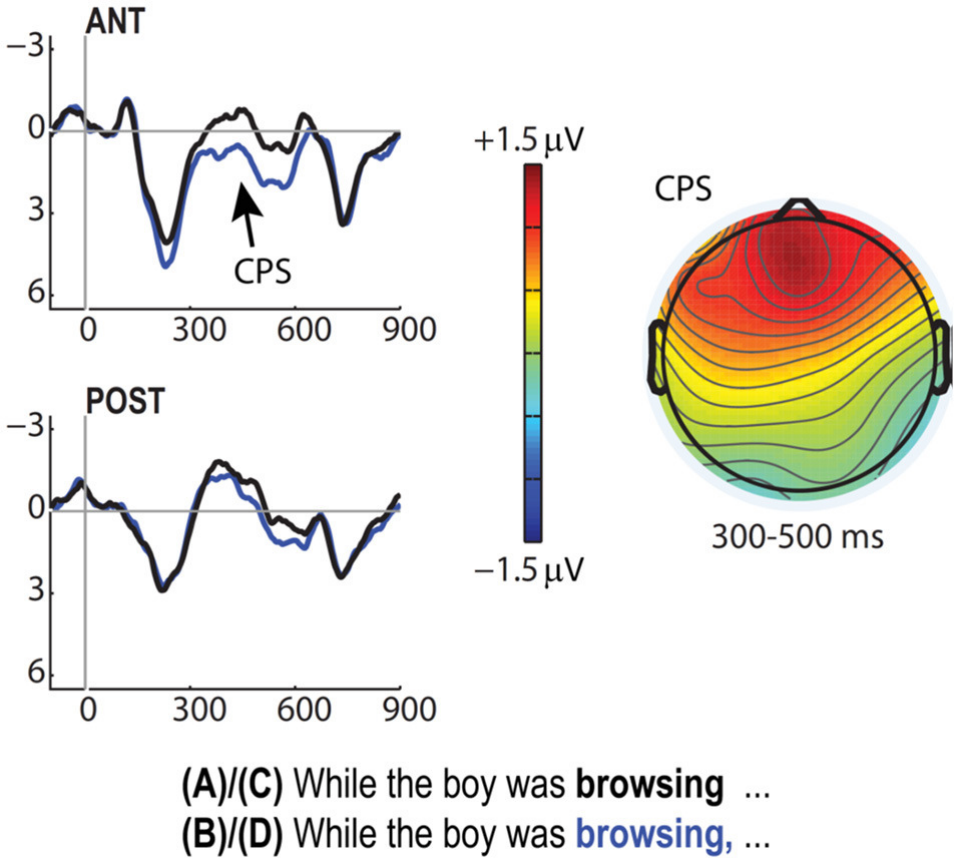
**CPS effect for the Closure ambiguity conditions, time-locked to the onset of the verb in the preposed adverbial clause (“browsing”)**. Grand average ERPs for anterior and posterior ROIs contrast comma-present conditions B/D (blue) vs. comma-absent conditions A/C (black). Voltage map shows mean amplitude differences over all scalp electrodes for the CPS (comma-present minus comma-absent); scale represents +1.5 μV (red) to −1.5 μV (blue).

Our closure ambiguity paradigm elicited several effects of interest. First, as with the parenthetical conditions, the presence of commas triggered a CPS (Figure 3), which was significant only the 300–500 ms time-window [*F*_(1, 25)_ = 5.69, *p* = 0.025], though it was marginal in the subsequent 500–700 ms range [*F*_(1, 25)_ = 3.65, *p* = 0.07]. Note that the issues concerning possible ambiguities in interpreting this response profile as a CPS (i.e., regarding P2 effects that arose for the commas in our parenthetical comparisons) do not arise here as there was no indication of any effects in the earlier 100–300 ms latency range.

**Figure 4:**
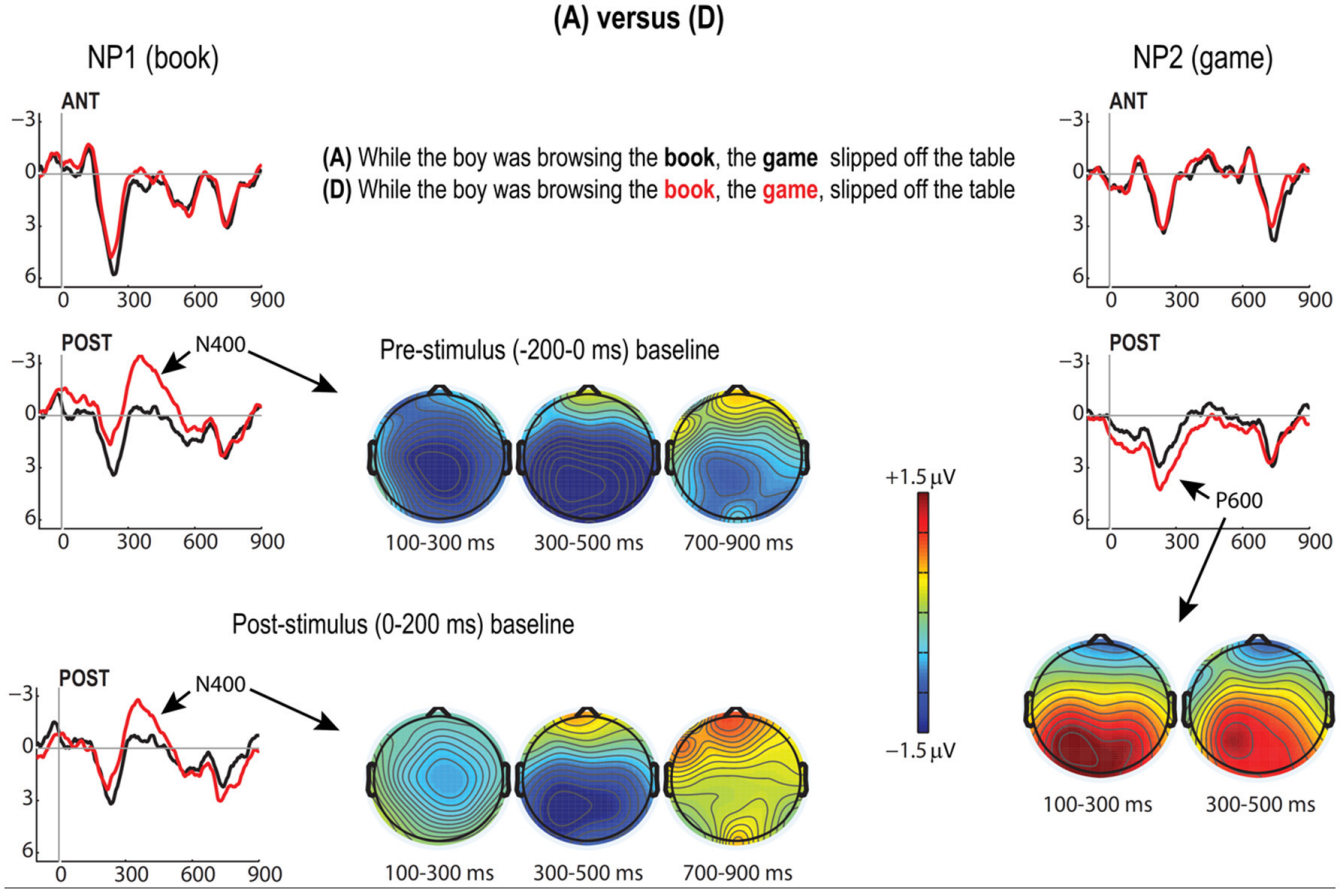
**N400 and P600 effects for the D vs. A contrast in the Closure ambiguity conditions**. Grand average ERPs (*N* = 26) plotted for anterior and posterior ROIs time-locked to the onset of the first post-verbal noun (NP1; “book”) on the left, and time-locked to the second noun (NP2; “game”). Baseline noise evident in the early latency ranges in the posterior ROI for NP1 led us to further examine this effect with an alternative (post-stimulus onset) baseline correction (0–200 ms). The N400 effect is shown in voltage maps for both the pre- and post-stimulus baseline corrections (left). Bottom right voltage maps show the subsequent P600 effect which emerged at the onset of NP2 (D minus A). Scale in these and all other voltage maps represents +1.5 μV (red) to −1.5 μV (blue).

Second, following Pauker et al. (2011), we expected that right after presentation of the first noun phrase in our (D) condition (*the book*), which is both preceded and followed by a comma and was largely judged unacceptable by our participants, we should observe a biphasic N400/P600 response. These effects did in fact obtain (Figure 4), but two additional issues cloud the picture.

The first issue is the obvious presence of baseline noise (see the 0 ms departures of the (A) and (D) conditions over the posterior ROI in Figure 4). Though the N400 effect demonstrates the characteristic scalp distribution (central parietal) and peaks at ~400 ms, the evident baseline noise makes the onset of this effect difficult to discern (cf., Steinhauer and Drury, 2012). As such, with our pre-stimulus baseline, this effect was already significant in the 100–300 ms time-window [*F*_(1, 25)_ = 5.69, *p* = 0.025], and it persisted through the next two windows, where both Condition [300–500 ms, *F*_(1, 25)_ = 8.80, *p* = 0.007] and Condition × AP interactions obtained [300–500 ms, *F*_(1, 15)_ = 21.88, *p* = 0.0008; 500–700 ms, *F*_(1, 25)_ = 10.65, *p* = 0.003], reflecting the evident posterior distribution of the negativity.

However, despite the presence of the baseline noise, it seems reasonable to conclude that this is, in fact an N400 effect. In addition to its characteristic distribution and the timing of its peak, the effect survives a 0–200 ms post-stimulus baseline correction, yielding the same Condition × AP interaction [*F*_(1, 25)_ = 24.09, *p* < 0.0001] as obtains in with the pre-stimulus baseline (see alternative baseline correction plotted in Figure 4). Further, comparing the 100–300 and 300–500 ms time-windows directly using the pre-stimulus baseline demonstrates a highly significant Condition × Time-window × AP interaction [*F*_(1, 25)_ = 31.45, *p* < 0.0001].

The second issue which clouds the (A)/(D) comparison is that the expected late positivity (P600) did not emerge until the onset of the subsequent noun (i.e., *game*), yielding significant Condition × AP interactions in the two earliest time-windows [100–300 ms, *F*_(1, 25)_ = 15.08, *p* = 0.0007; 300–500 ms, *F*_(1, 25)_ = 8.51, *p* = 0.007] (see P600 coinciding with the onset of the word *game*, bottom-right of Figure 4). Note that this effect is arguably best understood as the correspondent of the P600 seen in Pauker et al. (2011), and not (e.g.,) as a CPS effect elicited by the preceding comma, for three reasons. First, this positivity shows the characteristic posterior distribution of P600 effects (including other P600 effects found in the present study, see above), whereas the CPS effects in the present study have uniformly exhibited more fronto-central scalp distribution. The second reason is the timing: the effect we are labeling a P600 here had its onset after readers encountered the second noun *game* (i.e., some 1200 ms after encountering the preceding comma), whereas CPS effects in comma studies are always elicited within 300–500 ms after the words carrying the commas (here: the noun *book*). Thirdly, recall that *both* the violation condition D and its control condition A in Figure 4 contain a comma in this position (attached to *book*), such that the ERP difference between them cannot be due to this local comma but must reflect the processing difficulties due to the presence of a superfluous *early* comma in condition D (i.e., the first comma attached to *browsing*). To summarize, condition D elicited the expected P600, but this effect occurred later than in auditory versions of this experiment (e.g., Pauker et al., 2011).

**Figure 5:**
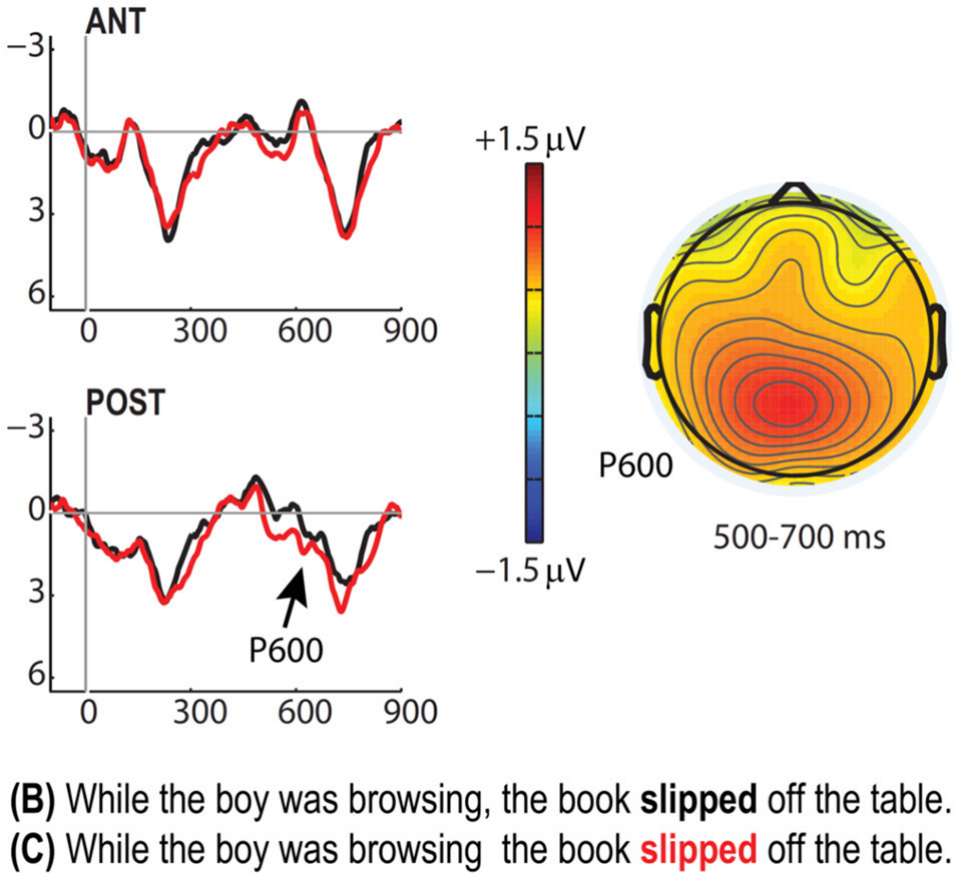
**P600 “garden path” effect for the C vs. B contrast**. Grand average ERPs (*N* = 26) for both anterior and posterior ROIs contrasting the main clause verb across the B (black) and C (red) conditions. Voltage map shows the P600 effect elicited for C relative to B [+1.5 μV (red) to −1.5 μV (blue)].

Finally, the last effect we report here for the Closure ambiguity cases is the garden path effect (P600) that was expected to arise on the main clause verb for condition (C) relative to (B). As predicted, this effect also obtained (Figure 5), demonstrating the characteristic timing and scalp topography of these responses [500–700 ms, Posterior ROI: *F*_(1, 25)_ = 4.41, *p* = 0.046].

## Discussion

The present study investigated the role of commas in two types of paradigms during silent reading, and used ERPs for the first time to reveal the online processing of punctuation marks in written English. We found that commas elicited CPS effects and had a dominating influence on parsing decisions for otherwise ambiguous input strings.

The seminal findings of CPS effects in silent reading of German (Steinhauer, 2003) suggested an important mechanistic role for our “inner voice” during visual processing of human language. However, until the present study it was unclear the extent to which these effects were general and replicable cross-linguistically. Here we showed reading CPS effects to be both. Not only do they obtain in English readers, they appear to do so in similar ways across very different functional uses of commas (i.e., serving to mark the boundary between a modifying clause and a matrix sentence vs. indicating the borders of parataxis).

### Commas and the CPS

As in previous ERP studies on comma processing in German and Chinese (Steinhauer and Friederici, 2001; Liu et al., 2010), but not in Dutch (Kerkhofs et al., 2008), commas at all positions and across both experiments elicited a fronto-centrally distributed CPS component in ERPs. In both sub-experiments, this positive shift occurred between 300 and 700 ms relative to the onset of the word marked with a comma. In line with previous research (e.g., Steinhauer, 2003), we interpret this positive shift as an electrophysiological marker for implicit prosody, specifically, the processing of a comma-induced prosodic break. This interpretation adds further credibility to current accounts of implicit prosody that assume that silent readers generally activate prosodic representations (Bader, 1998; Fodor, 1998, 2002). More specifically, our data confirm and extend Hirotani et al.'s (2006) conclusion that punctuation-induced wrap-up effects (reflected by longer reading times at punctuation marks) may be largely due to the processing of intonational phrase boundaries. Our present CPS findings demonstrate that silent readers of English sentences process these prosodic boundaries even if fixed presentation times (here: one word per 500 ms) do not permit them to slow down their reading pace.

However, given that the frontal topography of the CPS is similar to that of P200 onset and offset components, and since its latency overlaps with the offset P200 of the target word, one might argue that the larger positivity may simply reflect an increased P200. This concern appears plausible, because (1) the offset of a target word carrying an additional comma necessarily results in a larger visual contrast on the screen than the onset of the same word without a comma, and because (2) larger visual (and auditory) contrasts were found to elicit larger P200s (see Steinhauer, 2003). In fact, in a recent paper on musical phrasing, Glushko and colleagues have argued that the so-called ‘music-CPS’ found at musical phrase boundaries (e.g., Knösche et al., 2005) may be partly due to enhanced offset P200s, or to onset components of the following note (Glushko et al., 2016). Could a similar explanation be offered for the positivities reported here?

We believe a similar interpretation is not likely for the present CPS data, for the following reasons. *First*, if commas result in a larger P200s due to a stronger visual contrast, this effect should also occur during the *onset* of the comma-carrying word (i.e., when it replaces the blank screen). However, no systematic increase of the onset-P200 was observed, at least not for the commas in our closure conditions (but see Section Parentheticals and Gender Mismatches above). *Secondly*, while the latency of the CPS overlaps with the offset P200 (which clearly starts after 400 ms), the CPS in both experiments already begins around 300 ms, and thus too early for an offset P200. *Thirdly*, there is strong evidence from other silent reading studies that a CPS can be elicited in *absence* of a comma (or any other visual marker), if prosodic phrasing is induced by either mapping a prosodic contour on the written input (Steinhauer, 2003), or by creating long noun phrases in Korean that generally require a subsequent prosodic break (Hwang and Steinhauer, 2011). *Fourthly*, the various online garden-path effects in our data illustrate that commas had a major and immediate impact on parsing preferences that strongly resembled the impact of overt prosodic boundaries in speech (as in the Pauker et al., 2011 study). In other words, interpreting the CPS as a marker of implicit prosodic phrasing is the most parsimonious and most consistent account available.

Taken together, the CPS data and the behavioral data provide evidence that, on average, English readers strongly rely on commas in order to reach a sentence's (initial) interpretation. Thus, English readers seem to mirror the patterns previously found for German and Chinese readers (Steinhauer and Friederici, 2001; Liu et al., 2010), whereas the absence of CPS effects in Dutch readers is somewhat difficult to explain and certainly warrants further research. A limitation of our current study was that most participants were university students who can be argued to have above-average reading skills and likely a better command of punctuation skills as well. The German study by Steinhauer and Friederici (2001) showed that the amplitude and reliability of comma-induced CPS effects correlates with the consistency of punctuation in writing. This relationship has not yet been established in any other language, and differences in the composition of the tested sample of participants may underlie inconsistent findings across studies. However, given our main result that at least a substantial subset of English readers do use punctuation in real time while silently reading sentences, we might be able to use a similar ERP approach to study current controversies in English punctuation. For example, there has been some debate whether or not the so-called “Oxford comma” (or “serial comma”) between the last two items in certain coordination structures (e.g., the second comma in “Peter, Mary, and John”) should be removed from English punctuation rules (e.g., “Writer's Relief” website^2^; Truss, 2003). It should be possible to empirically demonstrate, whether and under which circumstances this comma helps to clarify meaning (i.e., beyond pure “intuitions” typically referred to in this controversy).

### Parentheticals and gender mismatches

Studying the impact of commas in distinguishing between sentence complements (*John said Mary was the nicest girl/?boy*) and parentheticals (*John, said Mary, was the nicest ?girl/boy*) provided an opportunity to completely cross the presence vs. absence of commas with semantic plausibility in a 2 × 2 design. Both offline acceptability data and online ERP data demonstrated that commas reversed the readers' parsing preference.

#### Sentences without commas

Without commas, readers analyzed the syntactic structure as a sentence complement, and accepted continuations with a plausible gender marking in 88%, while they rejected implausible ones in 76%. Although, these ratings still differ from a near-perfect performance expected from native speakers, the overwhelming preference in the judgment data suggests our manipulation generally worked as intended. As expected, in ERP online data, a robust N400 on implausible disambiguating gender-marked nouns (i.e., *girl/boy*) illustrated that mismatches with this structural analysis were immediately detected.

#### Sentences with commas

Importantly, in sentences containing commas, our participants showed a clear reversal of their syntactic online analysis in favor of a parenthetical reading. Here, disambiguating gender-marked nouns that were plausible with a complement reading but implausible with a parenthetical reading, elicited an N400, whereas those that had elicited this ERP pattern without commas did not. In other words, punctuation immediately changed the parsing preference before the disambiguating noun was reached. The presence of commas converted a plausible sentence into an implausible one, and vice versa. A limitation of this pattern was that, overall, sentences containing commas seemed to have caused more problems in determining the actual meaning. That is, well-formed (plausible) parentheticals were accepted only in 67%, and implausible parentheticals were correctly rejected in only 62% of the trials—both numbers are smaller than the corresponding ones for sentences that did not contain commas. We believe that the reason has to do with (a) the lower frequency and, likely related to this, (b) the more complex syntactic structure of parentheticals compared to clausal complement structures. Interestingly, violations in parenthetical constructions (containing commas) elicited an additional sentence-final N400-like ERP effect that was absent for our no-comma cases. Similar sentence-final negativities have previously been reported for a number of linguistic anomalies (Hagoort, 2003), including garden-path sentences (e.g., Osterhout and Holcomb, 1992), and are taken to reflect final plausibility checks. Applied to our sentences, this seems to suggests that readers may have been less confident about an implausible sentence interpretation (e.g., John being the nicest girl) if this interpretation depended on commas. They saw a need to “double-check” their initial interpretation (eliciting the sentence-final negativity) and sometimes even seemed to have changed it (resulting in the rather low rejection rate). If these challenges are more directly related to comma processing rather than to the syntactic complexity of parentheticals *per se*, an auditory replication of this study with overt prosodic boundaries should result in less ambiguity—and potentially prevent the sentence-final N400 effect as well.

Sorting this out, however, may depend on a firmer understanding of the relationship between parenthetical material and its host. Though the nature of such relationships in general is topic of ongoing research (Dehé, 2014), the sentence-final confusion in our comma conditions indicates that the linearly intervening proper name within the parenthetical can play an interfering role in resolving the gender-matching/predication relationships. Our cases may thus also be of interest in comparison to various types of “grammatical illusions” (Phillips et al., 2011), where elements that are structurally prohibited from entering into certain linguistic dependencies nonetheless appear to intrude in processing. For example, agreement attraction effects present such a situation, where intervening plurals interfere with subject-verb agreement (e.g., *the key to the cabinets* IS/?ARE…) in both language production (Bock and Miller, 1991) and comprehension (Wagers et al., 2009). The environments tested here would be valuable to examine in connection with this literature (e.g., *The boy, said the girls*, IS/?ARE…), since to our knowledge the question of how elements *within* a parenthetical may or may not interfere with the processing of dependencies between other elements *outside* the parenthetical has not yet been investigated.

### Garden path effects in closure ambiguities

The behavioral and ERP data found for closure ambiguities almost exactly replicate our previous findings in an auditory version of the same experiment (Pauker et al., 2011; see also Itzhak et al., 2010).

#### The P600 in condition C

For closure ambiguities, the literature suggests a strong preference in favor of late closure interpretations, such that the second noun phrase [NP; e.g., *the book* in Example (2) above] is interpreted as the direct object of the preceding verb (Frazier and Rayner, 1982; Frazier, 1987). However, a prosodic break between the verb and the NP can successful prevent this attachment and facilitate early closure processing (Kjelgaard and Speer, 1999; Pauker et al., 2011; see also Itzhak et al., 2010).

In the Early Closure conditions B and C, we found evidence that the “prosodically” disambiguating comma in condition B facilitated the processing. The likely mechanism is that the comma (and the associated prosodic break) detached the post-verbal noun phrase [e.g., *the book* in Example (2) above] from the preceding verb and prevented its interpretation as an object noun phrase (NP). As discussed in Pauker et al. (2011), in spoken language, English speaker would mark this position with an overt prosodic break, and English writers are expected to insert a comma in this position. If this boundary or comma is missing, the noun phrase tends to be interpreted as the direct object of the preceding verb (Late Closure principle; Frazier, 1987), such that the subsequent clause lacks a subject NP. This was the case in condition C, for which we found a reliable P600 garden-path effect on the lexically disambiguating verb (“*slipped*” in Example 2; see Figure 5). Thus, in absence of counter-evidence (from a comma), our readers seemed to have shown the typical preference for late close parsing, and had to revise the structure of condition C from late to early closure. The P600 effect for condition C relative to condition B was comparable to that found in the auditory study by Pauker and colleagues, suggesting that the disambiguating strength of a comma is comparable to that of a prosodic boundary.

The similarity across visual and auditory modalities is further supported by the behavioral data. Condition C was accepted at 47%, while condition B was accepted at 81% (Pauker et al. 53 and 87%, respectively). The reason why condition C, despite the P600 indicating processing difficulties, was still accepted in approximately half of the trials, can be accounted for with the ease of the required reanalysis. As discussed in various recent studies in the context of the *boundary deletion hypothesis* (BDH), the *post-hoc* creation or insertion of a prosodic boundary that was absent in the original input may be a relatively easy type of revision, whereas the *post-hoc* deletion of positive prosodic evidence (e.g., a speech boundary or a comma) is more effortful (Steinhauer and Friederici, 2001; Hwang and Steinhauer, 2011; Pauker et al., 2011; Bögels et al., 2013; Zahn and Scheepers, 2015).

#### N400 and P600 in condition D

Since condition D contains a superfluous early comma, the BDH would predict that this condition should be very difficult to reanalyze and, therefore, should result in very low acceptability ratings. As expected, our participants accepted this condition in only 35% of the trials, which was significantly lower than in condition C and, again, replicated the results of the auditory version, where the rate of 28% was even slightly lower (Pauker et al., 2011). In addition to comparable behavioral offline judgments between the two studies, we also observed similar online ERP effects. Unlike in condition C, for the more severe prosody-syntax mismatch in condition D we found both an N400 and a P600 effect. We adopt Pauker et al.'s interpretation of the same pattern, according to which the N400 reflects difficulties in assigning a theta role to the prosodically isolated (stranded) noun phrase, whereas the P600 is likely to comprise a number of cognitive processes, including difficult attempts to revise the structure. Overall, the striking similarities between the auditory and the reading version of this study confirm our hypothesis that commas and prosodic boundaries serve virtually identical functions in the two modalities, i.e., to provide early prosodic disambiguation in structurally ambiguous sentences. The lower acceptability and stronger ERP garden-path effects in condition D (compared to C) in both modalities support the predictions of the BDH.

There is one single finding in our ERP data that may suggest slight processing differences between the auditory and the visual version of the closure study. For all populations tested in the auditory modality, condition D consistently elicited its P600 effect right after the N400, virtually superimposing the CPS effect at the end of the first post-verbal noun phrase (e.g., *the book*) that was both preceded and followed by a prosodic boundary (Steinhauer et al., 2010; Pauker et al., 2011; Nickels et al., 2013; Nickels and Steinhauer, 2016). The interpretation in all of these studies was that the prosodically detached noun phrase caused both semantic and syntactic integration problems and was sufficient to initiate both the grammaticality judgment and possible attempts to repair the sentence structure (thereby eliciting the P600). In contrast, in our present reading study, the P600 in condition D was clearly triggered by the subsequent second noun phrase (i.e., *the game*)^3^. This apparent latency difference between the two modalities is compatible with two distinct interpretations. First, it is possible that in both modalities the same processing took place, i.e., the P600 was primarily triggered by the incoming second noun phrase, even in the auditory modality. However, whereas the fixed word presentation times in our reading study allowed us to clearly separate the CPS, the N400 and the P600 components, variability in auditory word durations—along with notorious baseline issues and a rather long duration of ERP components in the auditory studies (see Pauker et al., 2011)—may have rendered such a separation difficult for the auditory ERPs. Secondly, the P600 latency differences across modalities may be real and suggest that readers (unlike listeners) required additional lexical confirmation that the stranded first noun phrase *the book* was in fact not followed by a verb (and could be interpreted as an early closure sentence such as *When the boy was browsing, the book, slipped off the table*)^4^. Both interpretations underline the importance of replicating prosody-related ERP studies in the visual modality and provide a direction for future ERP studies in this domain. Importantly, however, the superfluous (early) comma in condition D (compared to A) resulted in the same sequence of ERP components (N400 and P600) as did the superfluous (early) prosodic boundary in the auditory version of condition D (Pauker et al., 2011).

In sum, given that commas reliably elicited a CPS component and replicated effects of overt prosodic boundaries in our study, we believe that the present data add credibility to the notion that commas serve as orthographic markers that induce implicit (subvocal) prosodic breaks in silent readers. In other words, it is this shared prosodic level of representation that results in the striking similarities across modalities.

## Author contributions

JD oversaw implementation of the design, including stimulus generation and randomization, programmed the scripts for stimulus presentation, carried out data analysis and interpretation, and wrote large parts of the manuscript. SB contributed the initial conceptualization and design of the study, contributed to data interpretation, and wrote parts of the manuscript. HV assisted in stimulus creation, collected most of the EEG data, and wrote parts of the manuscript. KS designed the study, oversaw the project as a whole, carried out ERP data analysis, interpreted the data, and wrote large parts of the manuscript.

## Funding

This research was funded through grants awarded to KS by the Canada Research Chair program (project # 950-209843), the Canada Foundation for Innovation (project # 201876), the Canadian Institutes of Health Research (project # MOP-74575), and the Social Sciences and Humanities Research Council of Canada (project # 435-2013-2052).

### Conflict of interest statement

The authors declare that the research was conducted in the absence of any commercial or financial relationships that could be construed as a potential conflict of interest.

## Footnotes

^1^The relationship between prosodic phrasing and syntactic structure in online processing is debated in several different sub-domains of sentence processing research. The IPH differs from other accounts based on studies of overt prosody (e.g., Clifton et al., 2006) in claiming that prosodic boundaries exhibit systematic influences on parsing decisions regardless of the types of cues that induce them. In contrast, Clifton et al. suggest that prosody may have limited impact on processing in certain situations, in particular where prosodic constituency is cued by phrase length (see Hwang and Schafer, 2009 for discussion). The IPH has also been appealed to as a way to understand cross-linguistic differences in the preferences found for resolution of attachment ambiguities involving relative clauses (e.g., whether the relative clause (RC) modifies the first or second noun phrase in cases like: *The servant of the actress* [_RC_ *that was on the balcony*], see Cuetos and Mitchell, 1988). However, though the IPH may be correct in claiming that prosodic differences between languages play a role in this domain, it is also clear that the IPH cannot serve as a general account of cross-linguistic variation in attachment preferences, and that there are other crucial factors involved (see Grillo and Costa, 2014). See also Breen (2014) for review discussion.

^2^Writer's Relief website on “the Oxford comma controversy”; http://writersrelief.com/blog/2014/07/oxford-comma-controversy/, accesses 01 May 2016.

^3^The P600 onset latency (around 100–200 ms after onset of *game*; see Figure 4) indicates that it was elicited by the preceding determiner, i.e., at the onset of “*the game”*); this corresponds to a prototypical P600 latency in reading studies of 600–700 ms (relative to the determiner “*the”*).

^4^This second interpretation might either point to saliency differences between speech boundaries and commas or suggest that processing commas in other conditions (i.e., in the parentheticals, which were not part of the auditory studies) may have affected the processing of commas in closure ambiguities.
